# Health-related quality of life of irritable bowel syndrome patients in different cultural settings

**DOI:** 10.1186/1477-7525-4-21

**Published:** 2006-03-27

**Authors:** Åshild Faresjö, Foteini Anastasiou, Christos Lionis, Saga Johansson, Mari-Ann Wallander, Tomas Faresjö

**Affiliations:** 1 Social Medicine and Public Health Science, Dept of Health and Society, Linköping University, Linköping, Sweden; 2 Clinic of Social and Family Medicine, School of Medicine, University of Crete, Greece; 3 Cardiovascular Institute, University of Gothenburg, Gothenburg, Sweden; 4 Dept of Public Health and Caring Science, Uppsala University, Uppsala, Sweden; 5 Dept of Epidemiology, AstraZeneca R&D, Mölndal, Sweden; 6 General Practice and Primary care, Dept of Health and Society, Linköping University, Linköping, Sweden

## Abstract

**Background:**

Persons with Irritable bowel syndrome (IBS) are seriously affected in their everyday life. The effect across different cultural settings of IBS on their quality of life has been little studied. The aim was to compare health-related quality of life (HRQOL) of individuals suffering from IBS in two different cultural settings; Crete, Greece and Linköping, Sweden.

**Methods:**

This study is a sex and age-matched case-control study, with n = 30 Cretan IBS cases and n = 90 Swedish IBS cases and a Swedish control group (n = 300) randomly selected from the general population. Health-related quality of life, measured by SF-36 and demographics, life style indicators and co-morbidity, was measured.

**Results:**

Cretan IBS cases reported lower HRQOL on most dimensions of SF-36 in comparison to the Swedish IBS cases. Significant differences were found for the dimensions mental health (p < 0.0001) and general health (p = 0.05) even after adjustments for educational level and co-morbidity. Women from Crete with IBS scored especially low on the dimensions general health (p = 0.009) and mental health (p < 0.0001) in comparison with Swedish women with IBS. The IBS cases, from both sites, reported significantly lower scores on all HRQOL dimensions in comparison with the Swedish control group.

**Conclusion:**

The results from this study tentatively support that the claim that similar individuals having the same disease, e.g. IBS, but living in different cultural environments could perceive their disease differently and that the disease might affect their everyday life and quality of life in a different way. The Cretan population, and especially women, are more seriously affected mentally by their disease than Swedish IBS cases. Coping with IBS in everyday life might be more problematic in the Cretan environment than in the Swedish setting.

## Background

Irritable bowel syndrome (IBS) is a functional disorder of the gastrointestinal tract and a quite common digestive disease in the general population frequently diagnosed in general practice [1]. IBS is widespread in all societies and socio-economic groups. For most patients, it is a chronic condition often with severe consequences [2]. There is strong evidence in previous studies that persons with IBS reveal impaired health-related quality of life [3-5]. Although this disease is not life threatening, patients with IBS seem to be seriously affected in their everyday life [6-9].

In assessing the impact of a (chronic) disease such as IBS on sense of wellbeing and daily functioning, patient-centred outcome data of health-related quality of life (HRQOL) are essential [10-12]. Previous studies of the impact of IBS on quality of life have either used generic health-related quality of life measurements, such as SF-36, or IBS-specific HRQOL instruments [9,13-15]. Disease-specific measures are especially used in clinical trials, while generic HRQOL measures are designed to evaluate aspects that are applicable to a population and therefore can provide a basis for comparisons with data from the general population [9,16].

A similarity concerning IBS patient's reports of their symptoms has been revealed in the sense that the patterns of GI symptoms seem to be similar across the Western cultures [17]. But, how are these symptoms and discomforts perceived by those affected? What is the impact on quality of life in different cultural settings? Are there any cultural differences in this respect? In a comparative study of HRQOL between the UK and the US, it was found that IBS had a significant impact on quality of life in both countries, but that this impact appeared to be greater in the UK than in the US [2]. In a study in the US of racial differences in relation to IBS, similar HRQOL was found between white and non-white IBS patients [18]. In general, some research suggests that cultural differences have an impact on the daily activities and quality of life of the IBS patients, but this has not been studied extensively.

The aim of this study was to use the SF-36 questionnaire to compare health-related quality of life of individuals suffering from irritable bowel syndrome in two different European cultural settings.

## Methods

### Study design

The design of this study is a matched case-control study, with two different groups of cases, IBS cases from rural and semi-rural villages on Crete, Greece, and IBS cases from the city of Linköping, Sweden. The criteria for identifying the cases and creating the databases were the same in the Greek and the Swedish settings. In primary care, the severity of the IBS disease could vary from mild and moderate to severe. In addition to the identified cases, a Swedish control group of non-IBS cases was randomly selected from the general population in the same Swedish region.

### The Greek group

Thirty cases with a diagnosis of IBS in the age groups between 17 and 65 years were identified through medical records at three health care centres on Crete. These 30 IBS cases are all actual cases in the age-group 17–65 years from a previous established IBS database with cases identified in a four-year retrospective survey of gastrointestinal problems in the population on Crete, which is reported elsewhere [19]. A medical doctor invited these 30 IBS cases to participate in an interview concerning health-related quality of life (the SF-36 questionnaire), demographics, life style indicators, gastrointestinal and other co-morbidity.

### The Swedish group

The Swedish IBS cases and control group were matched for gender and age with the Cretan IBS cases. Each Cretan IBS case was matched following the data collection with three Swedish IBS cases (3:1) and with 10 Swedish control group (10:1) from the general population. The Swedish IBS cases and control group were randomly selected from a large, previously established database consisting of N = 723 IBS cases and N = 4500 individuals from the general population. This database is based on the results of a five-year retrospective survey of diagnosed IBS cases identified through medical records at three health care centres in the city of Linköping located in the south-east region of Sweden [20]. In this study, a postal questionnaire, including SF-36, demographics, lifestyle indicators, gastrointestinal and other co-morbidity were used. The questionnaire was also sent to a random sample of the general population in the same region. The response rate was 71% for the IBS cases and 63% for the general population.

### Data collection

The same version of the generic health-related quality of life measure Short Form 36 (SF-36) was used in its Greek and Swedish translated form in this study. This instrument is well established and has been used extensively used in public health studies, epidemiology as well as in clinical trials [21,22]. The SF-36 includes eight multi-item scales that evaluate the extent to which an individual's health limits his or her physical, emotional and social functioning: physical functioning (10 items), role limitations caused by physical health problems (4 items), role limitations caused by emotional health problems (3 items), social functioning (2 items), emotional wellbeing (5 items), pain (2 items), energy/fatigue (4 items), and general health perceptions (5 items). All questions asked concerned the previous four weeks, with the exception of an additional item that assesses change in the respondent's health over the preceding year. Responses to the SF-36 were transformed into a standard scale ranging from 0, the worst possible score, to 100, the best possible score [23].

In addition to the HRQOL instrument, the subjects on Crete and in Sweden answered questions concerning demographics such as educational level and civil status. Additionally, some life style indicators such as smoking habits (daily smoker vs. non-smoker) were measured. In the group non-smokers ex-smokers could also be included. The variable insomnia was based on a question of how often the respondent felt they had had difficulty in falling asleep in the evenings. Those who reported that they sometimes, very often or always suffered from insomnia were regarded as having insomnia. The variable "perceived daily stress" was based on a question about how the respondent experienced daily stress. Data on co-morbidity were collected in the form of self-reports and focused on past or present occurrence of gastrointestinal diseases and chronic diseases. Gastrointestinal co-morbidity measured was: reflux, gastroenteritis, known peptic ulcer and other gastrointestinal complaints. Co-morbidity of other, mainly chronic, diseases measured was: coronary heart diseases, high blood pressure, diabetes mellitus, asthma, allergy, rheumatoid arthritis and depression.

### Ethics

The study was approved by the Research Ethics Committee of the Faculty of Health Sciences, Linköping University, Sweden and the Research Ethics Committee of the University Hospital of Heraklion, Crete, Greece.

### Statistical methods

All data were stored in a common database and statistically analysed using the SPSS 13.0 programme (SPSS Inc., Chicago, IL, USA). Significance of differences between cases and control group for SF-36 scale was estimated using the 2-sided ANOVA test. The SF-36 comparisons between Cretan IBS cases and Swedish IBS cases were adjusted in multiple regressions for significant differences in the distribution of the variables; educational level and co-morbidity regarding coronary heart diseases, high blood pressure, rheumatoid arthritis and depression. For categorical variables, the chi^2 ^test was used and when expected frequencies fell below five, Fisher's exact test was applied. In general, a p-value of < 0.05 was considered statistically significant.

## Results

The total of 420 participants in this study consist of n = 30 Cretan IBS cases, n = 90 Swedish IBS cases and n = 300 Swedish control group. The Swedish cases and control group were matched for gender and age with the Cretan cases. The ages of the cases and controls were distributed in the age groups as follows; 18–24 years: 3.3% (n = 14), age-group 25–44 years: 26.7% (n = 112) and age-group 45–64 years: 70.0% (n = 294). The gender distribution was 76.7% (n = 322) female and 23.3% (n = 98) male.

A comparison of some demographical data and life style indicators is presented in Table 1. The educational level of the Cretan IBS cases was significantly lower (p < 0.0001) than the Swedish IBS cases and control group. There were no significant differences in civil status between the groups. The number of full-time or part-time workers was significantly higher among the Swedish cases and control group in comparison with the Cretan IBS cases. The number of daily smokers was significantly higher among the Cretan IBS cases than among the Swedish cases and control group. Insomnia was most common among the Swedish IBS cases and also higher among the Swedish control group in comparison with the Cretan IBS cases. A significantly (p < 0.0001) larger proportion of the IBS cases from both Crete and Sweden experienced daily stress often or very often in comparison with the Swedish control group.

**Table 1:** Comparison of demographically data and life style indicators between Cretan and Swedish IBS cases and between all IBS cases (from both sites) and Swedish control group

	**Cretan IBS Cases (n = 30)**		**Swedish IBS cases (n = 90)**			**Swedish control group (n = 300)**		
	**n**	**%**	**n**	**%**	**p**	**n**	**%**	**p**
**Educational level**					< 0.0001			< 0.0001
Primary (low)	19	63.3	18	20.0		64	21.4	
Secondary	6	20.0	23	25.6		68	22.7	
High school	4	13.3	16	17.8		54	18.1	
College/University (High)	1	3.3	33	36.7		113	37.8	
**Marrital status**					0.14			0.20
Single	1	3.3	10	11.2		36	12.1	
Married or cohabiting	21	70.0	67	75.3		225	75.5	
Divorced or widow	8	26.7	12	13.5		37	12.4	
**Occupational situation**					0.001			< 0.0001
Full or part-time	11	36.7	64	71.1		220	73.6	
Student, on sick leave or unemployed, etc	19	63.3	26	28.9		79	26.4	
**Smoking habits**					0.01			0.05
Daily smoker	8	26.7	8	8.9		43	14.7	
Non-smoker	22	73.3	82	91.1		249	85.3	
**Insomnia**					< 0.0001			0.001
Yes	7	23.3	55	61.1		143	48.3	
No	23	76.7	35	38.9		153	51.7	
**Experienced daily stress**					0.30			< 0.0001
Very often or Often	16	53.3	55	64.0		96	33.7	
Seldom or Never	14	46.7	31	36.0		189	66.3	

Reported previous or current gastrointestinal co-morbidity for the cases and control group is shown in Table 2. Previous or current gastrointestinal co-morbidity, with the exception ulcer, was significantly more frequently reported among the IBS cases in both locations than the matched Swedish control group. When comparing the two groups of IBS cases, previous GI complaints were significantly more frequently reported among the Swedish IBS cases. Among the Cretan IBS cases, there were more frequent reports of co-morbidity concerning coronary heart diseases (p = 0.036), high blood pressure (p = 0.021) and rheumatoid arthritis (p = 0.003) than among the Swedish IBS cases. Depression, on the other hand, was more frequently reported (p = 0.026) among the Swedish IBS cases than the Cretan IBS cases. There were no differences in the occurrence of co-morbidity such as diabetes mellitus, asthma and allergy between the Cretan and Swedish IBS cases.

**Table 2:** Reports of current and previous gastrointestinal co-morbidity between Cretan and Swedish cases and between all IBS cases (from both sites) and Swedish control group

	**Cretan IBS Cases (n = 30)**		**Swedish IBS cases (n = 90)**			**Swedish control group (n = 300)**		
	**n**	**%**	**n**	**%**	**p**	**n**	**%**	**p**
**Peptic ulcer**					0.19			0.18
Yes	4	13.3	5	5.8		14	5.0	
No	26	86.7	82	94.2		267	95.0	
**GI complaints**					0.002			< 0.0001
Yes	11	36.7	60	68.2		66	23.2	
No	19	63.3	28	31.8		219	76.8	
**Reflux**					0.28			< 0.0001
Yes	9	30.0	35	41.2		56	19.5	
No	21	70.0	50	58.8		231	80.5	
**Gastroenteritis**					0.07			< 0.0001
Yes	16	53.3	30	34.5		63	22.2	
No	14	46.7	57	65.5		221	77.8	

A general tendency was that the Cretan IBS cases reported lower HRQOL on six of the eight dimensions of SF-36 than the Swedish IBS cases, see Table 3. These differences were most evident in the dimensions general health and mental health. After adjustments in multiple regressions for the differences in the distribution of educational level and occurrence of present or past co-morbidity (coronary heart disease, high blood pressure, rheumatoid arthritis and depression), the Cretan IBS cases nevertheless scored lower in general health (p = 0.05) and lower in mental health (p < 0.0001) than the age and sex-matched Swedish IBS cases. A gender analysis revealed that Cretan women with IBS scored especially low on the dimensions general health p = 0.009 (mean score: 48.0 s.d: 20.3) and mental health p < 0.0001 (mean score: 48.6 s.d: 24.9) in comparison with Swedish women with IBS (general health mean score: 62.3 s.d: 23.2 and mental health mean score: 71.0 s.d: 16.3). When analysed together, the IBS cases from both countries reported significantly lower scores on all quality of life dimensions in comparison with the Swedish control group.

**Table 3:** Comparison of health-related quality of life (SF-36) between Cretan and Swedish IBS cases and between all IBS cases (from both sites) and Swedish control group

	**Cretan IBS Cases (n = 30)**		**Swedish IBS cases (n = 90)**			**Swedish control group (n = 300)**		
	**mean**	**sd**	**mean**	**sd**	P*	**mean**	**sd**	**p**
**Physical function**	73.7	30.4	83.9	21.4	0.57	88.9	15.2	< 0.0001
**Physical role**	75.0	43.1	71.8	35.9	0.21	84.9	28.9	0.002
**Bodily pain**	61.0	31.7	67.2	23.5	0.88	80.7	20.8	< 0.0001
**General health**	50.4	22.4	63.3	23.5	0.05	75.0	19.9	< 0.0001
**Vitality**	55.0	31.4	52.1	23.7	0.52	66.7	20.5	< 0.0001
**Social function**	74.6	36.0	77.5	25.5	0.52	89.4	17.6	< 0.0001
**Emotional role**	74.4	34.7	76.5	35.8	0.71	86.9	26.9	0.004
**Mental health**	50.0	26.0	72.1	17.1	< 0.0001	79.5	17.3	< 0.0001

*Adjusted in multiple regressions for; educational level and present or past co-morbidity of coronary heart diseases, high blood pressure, rheumatoid arthritis and depression.

## Discussion

It is known that persons with the common digestive disease IBS reveal impaired HRQOL [3-5]. However, the impact on quality of life for those affected in different cultural settings has not been studied extensively. The results from this study tentatively indicate that there are differences in how persons with IBS on Crete, Greece, and in Linköping, Sweden, perceive their disease and how it affects their quality of life. This is especially noticeable as regards impaired mental health and reduced general health, where the Cretan IBS cases reported a lower HRQOL, even after adjustments for differences in the distribution of educational level and co-morbidity. However, there was no significant difference between the locations concerning social functioning. Since all the IBS cases in this study are identified in Cretan and Swedish primary care, the severity of the conditions can thus be expected to be the same in both locations.

The hypothesis that the impact of IBS on HRQOL varies in different cultural settings has also been supported by an earlier study where IBS patients in the UK and the US were compared [2]. Analyses of health-related quality of life without any comparisons between cultural settings have previously been presented for other gastrointestinal disorders such as inflammatory bowel disease (IBD) and ulcerative colitis in both Sweden and on Crete in Greece [30,31]. However, these studies focused on other gastrointestinal disorders than IBS and the subjects were hospital out- and in-patients and not in primary care and the general population and are thus not comparable to the present study.

A plausible explanation of the differences found in this study is that coping with IBS in everyday life might be more problematic in the Cretan environment than in Sweden and this represents the main finding. The outdoor living tradition and the warm climate with long and hot summers together with a higher risk of gastroenteritis in combination with the IBS disease might negatively influence their everyday quality of life. The disease might possibly also cause a feeling of being out of the ordinary when affected by a quite sensitive and slightly embarrassing condition. This might partly explain why the Cretan IBS cases, and especially the Cretan women, scored significantly lower on the mental health dimension.

The IBS cases from both locations reported experienced daily stress significantly more often than the Swedish control group. The link between IBS and psychosocial factors such as stress in everyday life has been reported in many earlier studies [33,34]. An interesting finding in this study was that significantly more Swedish IBS cases and controls reported insomnia than did Cretan cases. Sleeping problems have been found to be associated with the IBS disease in other studies [35,36]. Daily stress as well as insomnia is associated with modern society, but the individual's perception of these phenomena might be varying between different cultural environments.

The Cretan IBS cases come from rural and semi-rural villages on Crete while the Swedish cases and controls come from urban areas in Sweden, which adds contrast to the cultural differences between the sites. This difference is also reflected in the variables educational level and full-time or part-time work. These socio-demographic differences between the sites might have had an impact on the results that increased the differences in self-reported quality of life. The dissimilarity in the way the data collection was carried out in the Cretan and Swedish sites, interview versus postal survey, might also have had some influence on the results, but it is doubtful as to how and to what extent. In comparative studies, using the same way of collecting the data is always preferable. However, postal surveys are not readily available as a data collection method on Crete and the probable response rate can thus be expected to be very low. In terms of local conditions on Crete, interviews are the best way of collecting data. On the other hand, as regards Swedish conditions, postal surveys are quite appropriate and cost-effective as a method of collecting population-based data.

A possible uneven distribution of different types of co-morbidity between the Cretan and the Swedish IBS cases might affect HRQOL. Although we made adjustments for some co-morbidity in the analysis, we cannot rule out the possibility that the Cretan cases might to some extent be affected by other unmeasured co-morbidity apart from IBS, which might lead to lower HRQOL. But Cretan inhabitants are considered to be one of the healthiest populations in Europe and have attracted considerable interest from a public health point of view [24-26]. For example, the traditional Mediterranean diet represents a healthy nutritional pattern [27]. Explanations that Cretans might be more affected by other serious co-morbidity not measured in this study are thus not so plausible. All cases and controls in this study are matched, so the differences found are not a consequence of either of gender or age-related ill health.

There was no control group available from the Greek location at the time of this data collection and this reduces to some extent the degree of comparability between the sites. Recently, some preliminary general population normative SF-36 data [28] have been published. However, these data are not quite comparable, i.e. not from the same geographical area as the cases in our study since they were collected in the city of Athens and not rural or semi-rural of Crete, and, further, the data were insufficient to form an age and sex-matched Greek control group in the analysis. In the present study, the control group had to solely be a Swedish age and gender-matched control group from the same geographical area as the Swedish IBS cases. Nevertheless, this study design with age and gender matched-controls has been recommended for optimal measurement of HRQOL outcomes of gastrointestinal diseases [29]. There might be a general culturally related difference between the two countries in the perception of quality of life. In a study of HRQOL, comparing a healthy Greek population with national norms in the general populations in US and several European countries, it was found that the mean scores on all SF-36 dimensions reported by the Greek participants were considerably lower than those in the other nations [32].

The findings in this study emphasise that perceptions of living conditions and quality of life must be interpreted in the light of cultural differences between these two European locations. Cultural differences between these two settings were observed in both working and social life in the local community. The role and importance of health behaviour and health beliefs, the social environment including family and religious beliefs are also other cultural factors to be considered. These aspects and their relationship with the perception of quality of life concerning IBS patients need to be further elaborated in future studies.

## Conclusion

The results from this study tentatively support the claim that similar individuals having the same disease, such as IBS, but living in different cultural environments could perceive their disease differently and that the disease might affect their everyday life and quality of life in a different way. Health planning interventions as well as medical treatment should take such findings into consideration, especially when models from another country are about to be adopted. These findings might also have implications for health planning, primary care management and clinical trials. Future studies comparing patients from different cultural environments will give a clearer picture of the real impact of IBS on quality of life.

## Competing interests

The author(s) declare that they have no competing interests.

## Authors' contributions

ÅF, FA, TF designed and coordinated this study. CL supervised the Greek data collection carried out by FA. ÅF performed all statistical analyses. ÅF, FA, TF, CL, SJ and M-AW interpreted the data and drafted and edited the manuscript. All authors read and approved the final manuscript.
